# Carcinome épidermoïde survenant sur une lésion d’ostéomyélite chronique: rapport de cas

**DOI:** 10.11604/pamj.2020.37.307.22388

**Published:** 2020-12-03

**Authors:** Aniss Chagou, Hamza Benameur, Najib Alidrissi, Mohammed Chahbouni, Ali Zine, Salim Bouabid, Moustapha Boussougua, Abdeloihab Jaafar

**Affiliations:** 1 Mohammed VI University of Health Sciences (UM6SS), Rabat, Maroc

**Keywords:** Carcinome épidermoïde, ostéomyélite chronique, transformation maligne, rapport de cas, Squamous cell carcinoma, chronic osteomyelitis, malignant transformation, case report

## Abstract

L´association d´un carcinome épidermoïde et d´une ostéomyélite chronique est rare. Elle doit cependant être évoquée à chaque fois qu´il y a une modification de la symptomatologie locale usuelle. Le retard du diagnostic peut être fatal pour le patient. Les biopsies répétées peuvent être d´une aide précieuse pour le médecin traitant. Nous rapportons le cas d´un patient de 47 ans chez qui un diagnostic de dégénérescence carcinomateuse sur une ostéomyélite chronique fistulisée à la peau a été posé. Une amputation a été réalisée.

## Abstract (en)

Squamous cell carcinoma associated with chronic osteomyelitis is rare. However, it must be suspected in patients with modification of common local symptoms. Delayed diagnosis can be fatal. Repeated biopsies can greatly help physicians who treat patients. We report the case of a 47-year-old patient diagnosed with carcinomatous degeneration associated with chronic osteomyelitis fistulized to the skin. Amputation was performed.

## Introduction

L´apparition d´un carcinome épidermoïde est une complication rare dans l´évolution d´une ostéomyélite chronique [1]. Peu de cas ont été rapportés jusqu´à présent dans la littérature. Si le mécanisme physiopathologique reste pour le moment inconnu, le délai important entre les premiers symptômes de l´ostéomyélite et la dégénérescence est un point commun de tous les cas rapportés.

## Patient et observation

**Figure 1:**
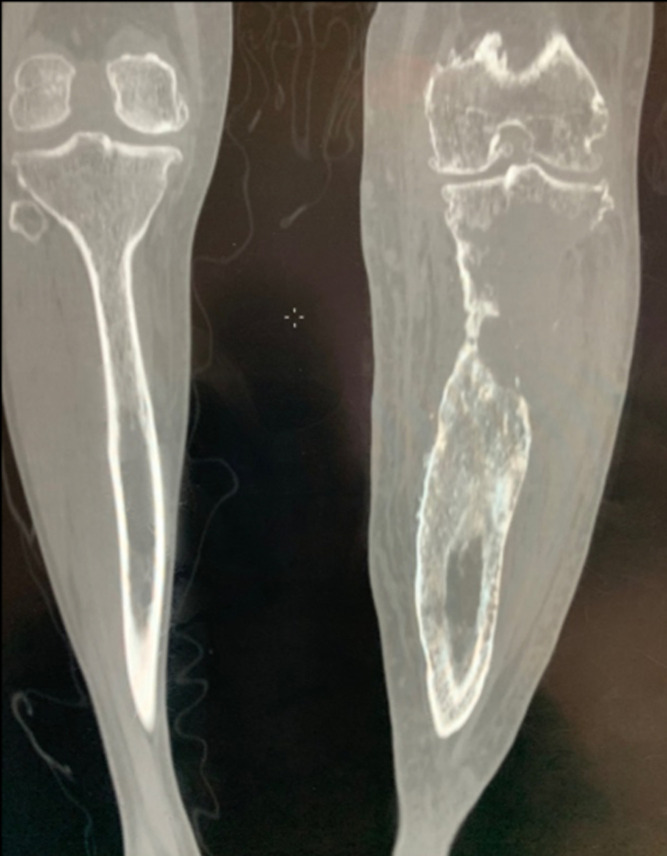
TDM de la jambe gauche en coupe frontale

**Figure 2:**
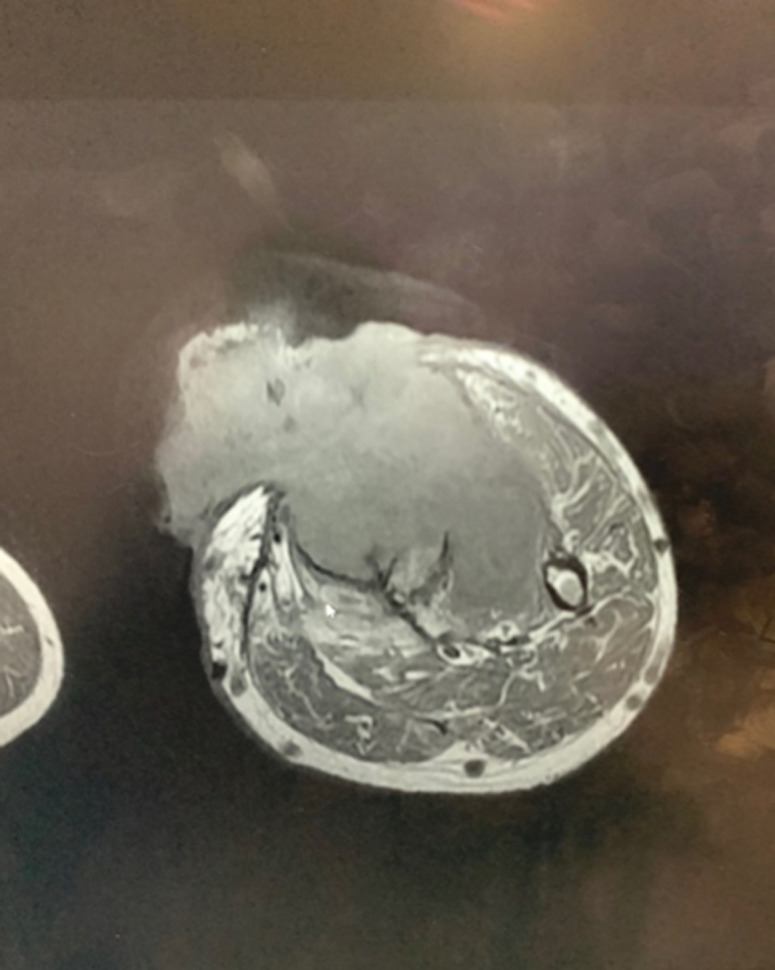
IRM de la jambe gauche en coupe transversale

**Figure 3:**
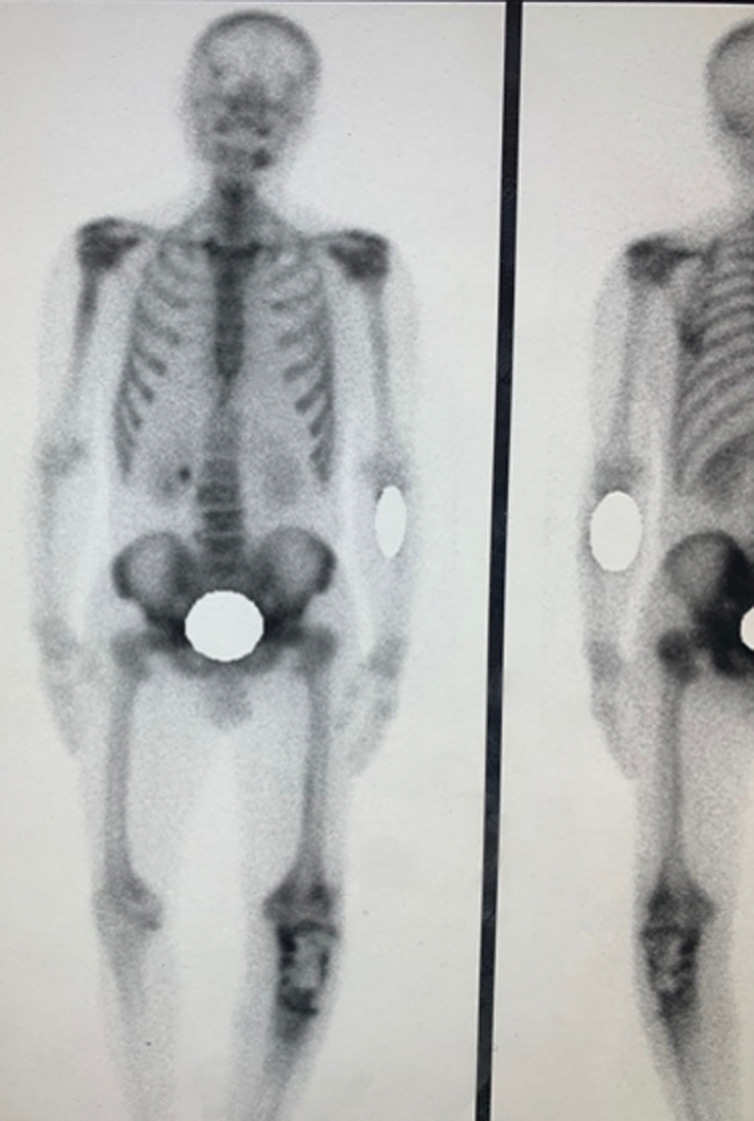
scintigraphie osseuse ; vue frontale (image de gauche) et vue dorsale (image de droite)

Nous rapportons le cas d´un patient de 47 ans, traité durant 7 ans pour une ostéomyélite chronique d´origine hématogène à l´âge de 7 ans. Le patient a bénéficié d´un traitement chirurgical type Papineau avec des résultats initiaux très satisfaisants. L´antibiothérapie a permis de contenir l´infection. Un seul épisode de réactivation a été noté à l´âge de 20 ans avec écoulement purulent. Une séquestrectomie avec excision du trajet fistuleux et des berges ont été réalisés. Une période de rémission de 27 ans a été notée. Le patient a été reçu dans notre département en mars 2018, le patient avait une grosse tumeur au niveau de la jambe au niveau du tiers supérieur volontiers saignante et malodorante. Le patient rapportait également une impotence fonctionnelle totale et un écoulement purulent. La tomodensitométrie (TDM) réalisée (Figure 1) a objectivé une lésion hétérogène associant des images lytiques extensives épiphyso-métaphyso-diaphysaires et hyperdenses avec effraction de la corticale. Le bilan sanguin a objectivé une anémie à 5g/dl. Une transfusion a été réalisée avant la biopsie. L´examen anatomopathologique a révélé des images d´une tumeur maligne en faveur d´un carcinome épidermoïde. Un bilan d´extension locorégionale fait d´une imagerie par résonance magnétique (IRM) (Figure 2) a révélé une tumeur d´allure agressive tissulaire avec envahissement osseux et vasculaire. Une scintigraphie (Figure 3) a montré une hyperfixation au niveau de la jambe gauche sans autre localisation. Une amputation trans-fémorale a été réalisée. Les résultats à ce jour sont bons.

## Discussion

La dégénérescence maligne au décours d´une ostéomyélite chronique est relativement rare. Sa fréquence varie de 0,2% à 1,7% selon les séries [1-3]. On note une nette prédominance masculine et un âge moyen assez élevé de 50 à 60 ans [1-4]. Ces carcinomes épidermoïdes ne représentent qu´une partie infime de tous les carcinomes épidermoïdes (0,05%) [5]. Les lésions siègent électivement au niveau des membres inférieurs (89%) surtout les jambes. Les lésions au niveau des membres supérieurs restent rares. Le délai entre le diagnostic de l´ostéomyélite chronique et la dégénérescence est habituellement long (37 ans) [5-7]. Dans notre cas, le délai est de 40 ans. Le retard du diagnostic de la transformation maligne est une constante. Les lésions apparaissant sur une peau déjà modifiée par l´infection chronique et la présence habituelle du pus n´attirent pas l´attention du patient et des médecins. Le diagnostic peut se faire des années après la transformation maligne. Un suivi régulier pourrait permettre d´évoquer le diagnostic. Rowland [8] a décrit trois symptômes devant faire évoquer le diagnostic: l´accentuation de la douleur et de l´écoulement purulent et surtout des lésions volontiers hémorragiques. Cette triade n´est que très rarement complète. Notre patient ayant consulté tardivement a présenté ces trois signes.

Devant toute suspicion, des biopsies répétées, au niveau des tissus superficiels et profonds doivent être réalisées [9]. Le traitement de ces lésions doit éradiquer à la fois la tumeur et l´infection: l´objectif étant d´éviter la récidive. Dans ce cas de figure, seule la chirurgie peut donner satisfaction. Si l´amputation permet l´éradication à la fois du foyer infectieux et tumoral; d´autres auteurs proposent un traitement conservateur moyennant une reconstruction osseuse et des parties molles [10,11]. Les récidives locales sont possibles 15% [12]. Les métastases le sont également avec un taux de 15 à 30% [3]. Chez notre patient, l´amputation trans-fémorale a été réalisée pour éviter le risque de métastases et de récidive locale [12]. A ce jour, aucune récidive ni métastases n´ont été diagnostiquées.

## Conclusion

La dégénérescence carcinomateuse dans les suites à long terme de l´ostéomyélite chronique est rare. Un suivi régulier permet d´éviter tout retard du diagnostic qui peut être fatal pour le patient. Devant toute modification de la symptomatologie, des biopsies répétées des tissus superficiels et profonds doivent être réalisées. La confirmation d´une transformation maligne impose la réalisation d´un bilan d´extension loco-régionale. La chirurgie doit garantir l´éradication du foyer infectieux et tumoral pour éviter la récidive.
